# Pollination Effectiveness of the Hoverfly *Eristalinus aeneus* (Scopoli, 1763) in Diploid and Triploid Associated Watermelon Crop

**DOI:** 10.3390/insects13111021

**Published:** 2022-11-05

**Authors:** Manuela Sánchez, Yelitza Velásquez, Mónica González, Julián Cuevas

**Affiliations:** 1 Department of Agronomy, University of Almería, ceiA3, Ctra. Sacramento s/n, La Cañada de San Urbano, 04120 Almería, Spain; 2 Department of Research and Development, Polyfly S.L., Avenida de la Innovación 15, 04131 Almería, Spain; 3 Experimental Station Foundation Cajamar, Paraje las Palmerillas, 25, 04710 El Ejido, Spain

**Keywords:** hoverfly, pollination, watermelon, *Eristalinus aeneus*, plant–pollinator interactions, alternative pollinators, myophily

## Abstract

**Simple Summary:**

Watermelon is an important crop worldwide. However, pollination is a challenge in new commercial seedless cultivars, exacerbated by the decline in pollinators and protected production systems. These negative factors affect yield due to pollination deficit. Therefore, a search for alternative pollinators is important, evaluating their pollination effectiveness. In this work, we studied the pollination of diploid and associated triploid seedless watermelon varieties using eristaline hoverflies, focusing on the characteristics of floral visits, pollen transport, and crop yield. We found that *Eristalinus aeneus* is an effective pollinator in watermelon at densities of 30 and 45 individuals per square meter.

**Abstract:**

Watermelon (*Citrullus lanatus*) is an important crop worldwide. Pollination of this crop is carried out by insects, with honey bees (*Apis* spp.) and bumble bees (*Bombus* spp.) as the most used in greenhouse production. Nevertheless, due to the extreme conditions in closed enclosures, these hymenopterans suffer management and behavior problems leading to insufficient pollination. The effectiveness of three release densities (15, 30, and 45 individuals/m^2^) of *Eristalinus aeneus* was compared in diploid- and triploid-associated watermelon varieties under protected cultivation. Floral visits, pollen–pistil interaction after pollen transport, yield, and fruit quality were evaluated. The number of floral visits increased with release density in both pistillate and staminate flowers. No significant differences were observed, however, among release densities or between flower types in the duration of the visits. Floral preferences were not found in the behavior of *E. aeneus* in watermelon. High and medium release densities increased pollen deposition onto the stigma, and consequently the yield of the triploid variety compared to low release density, by 23.8 to 41.8% in 2020 and by 36.3 to 46.7% in 2021. The results of this trial demonstrate the potential of *E. aeneus* as a managed pollinator in protected cultivation of triploid watermelon.

## 1. Introduction

Cultivated watermelon [*Citrullus lanatus* var. lanatus (Thurb.)] is a monoecious creeping herbaceous crop belonging to the family Cucurbitaceae. Watermelon is among the ten most economically important horticultural crops, with the cucurbit having the highest production worldwide [1,2]. This species is native to tropical Africa and well adapted to hot and dry climates necessary for fruit development and ripening [1]. The cultivation of watermelon under greenhouse conditions enables early harvesting, favoring spring production in Europe and the Mediterranean basin, thus advancing its commercialization in international markets. In addition, greenhouse cultivation increases yield and fruit quality due to the protection provided against harsh environmental conditions and the better control of pests and diseases. In Spain, the area of protected cultivation of watermelon has increased by 36% in the past ten years.

Commercial watermelon includes diploid (seed-bearing and mini fruits) and triploid (seedless fruits due to early seed abortion because of stenospermocarpy) varieties, the latter being the most sought-after fruits [3]. Despite the absence of seeds in triploid varieties, these cultivars require cross-pollination to set fruit. Watermelon bears pistillate (female) and staminate (male) flowers, in ratios varying from 1:7 to 1:17. Flower anthesis occurs in the morning and the flowers remain open for only 9 h [3]. The floral rewards present in watermelon are pollen in the staminate flowers and nectar in both, the pistillate and staminate flowers. Staminate flowers of triploid cultivars lack viable pollen grains [4], so it is always necessary to introduce a diploid variety as a pollen donor and a proper transportation of pollen from the staminate flowers of diploid cultivars to the pistillate flowers to set fruits. For this transport, watermelon depends entirely on insects [5,6]. According to Gallai [7], for fruit set, the insect pollinator dependence of this crop is between 90 and 100%. In addition, because of the multi-seeded condition of watermelon, optimal pollination requires between 500 and 1000 pollen grains well distributed on the stigma of the pistillate flowers to produce a commercial fruit [5,8,9]. Therefore, flowers with less than 500 pollen grains attached may not develop into commercial fruit.

Pollination in commercial watermelon crops is carried out by honey bees (*Apis* spp.) and, less often, by bumble bees (*Bombus* spp.). However, pollination deficits often occur due to their inadequate pollination service [10]. In this regard, watermelon production confined within a closed greenhouse makes it difficult for bees to forage. There, the extreme environmental conditions, the lack of food, the use of pesticides, and the stress of foraging in closed enclosures often cause unsatisfactory behavior of these hymenopterans [11,12,13,14,15,16,17]. Another limiting factor for bees in protected cultivation is the negative influence of photo-selective plastic on their vision, making it difficult for them to identify the flowers and orient themselves correctly [18,19,20]. Another factor that increases the pollination deficit is the difficulty of access by wild pollinators to the protected crop. Given the problems mentioned above, some authors have suggested the use of attractants to improve honey bees’ performance [21] and, alternatively, the use of plant growth regulators to set fruit [22]. These measures interfere with food safety, increase costs, and still do not obtain satisfactory yields. Despite these objections, only pollination plans based on hymenopterans such as bees and bumblebees have been developed in watermelon [23,24,25,26,27,28,29,30,31].

Although the honey bee stands out as the primary crop pollinator for watermelon worldwide, many other species contribute to watermelon pollination in the open field [24,27,30,32,33,34,35]. Moreover, the floral characteristics of watermelon (actinomorphic, i.e., radially symmetric and discoidal shape) and its pollination syndrome (entomophily, i.e., insect pollination) suggest using other group of insects as pollinators. In watermelon, dipterans have not yet reached the condition of managed pollinators, although they are cited as frequent visitors to their flowers [24,33,34,36]. Among the dipterans, syrphids stand out for their great potential as pollinators of crops and wild flora. This group, of which more than 6000 species have been described, belongs to the family Syrphidae. They are commonly known as flower flies or hoverflies, due to two peculiarities: their characteristic flight, rapid and sustained in the air, and their anthophilous character with constant floral visits. During the adult phase, most hoverflies show generalist foraging on flowers, visiting a wide variety of species to feed on nectar and pollen through the proboscis [37,38,39].

In this context, *Eristalinus aeneus* is a hoverfly well established in the Mediterranean basin and abundant during watermelon flowering season. Its pollination effectiveness has been verified in fruit and seed crops, showing great potential as a managed pollinator [40,41]. Therefore, the objective of this study was to determine the pollination effectiveness of *E. aeneus* by comparing yield and fruit quality under three release densities of this syrphid in protected cultivation of watermelon. In addition, their pollination activity and efficacy were studied through the number of floral visits and pollen transport to the stigma of the flowers. The final aim of this experiment was to determine the optimum release density of this syrphid for pollinating watermelon under protected cultivation.

## 2. Materials and Methods

### 2.1. Field Experiment Location and Biological Material

The trial was carried out at the UAL-Anecoop experimental farm, in a greenhouse located in the municipality of Retamar, Almería (Spain) (36°51′55″ N, 2°17′01″ W). The experimental greenhouse had a total area of 1024 m^2^, having a parral-type structure with eight chapels oriented E–W, provided with a three-layer polyethylene cover, and passive lateral ventilation with insect-proof mesh. The structure was 32 m wide and 32 m long, 3 m high in the eaves and 4 m in the ridge. The plantation was carried out with the commercial varieties Premium (a diploid, mini-type cultivar) and Fashion (a triploid, seedless cultivar), in a ratio of 1:3 and density of 0.25 plants/m^2^. The crop was managed according to the technical recommendations of the UAL-Anecoop Foundation personnel.

The trial was designed following a randomized block experiment with three treatments and four replications. The treatments were high density (HD) *E. aeneus* release specified as 45 individuals/m^2^, medium density (MD) with 30 individuals/m^2^, and low density (LD) with 15 individuals/m^2^. Within the greenhouse, a total of 12 white mesh cages of 6 × 6 threads/cm^2^ were built, with a surface of 32 m^2^ (4 × 8 m), each containing six triploid and two diploid watermelon plants. Inside each cage, a single release of pupae of the syrphid *E. aeneus* from the supplier Polyfly was made according to the treatment: 1440 pupae in HD, 960 pupae in MD, and 480 pupae in LD. Emergence of adult hoverflies started approximately 3 days after release into the crop and lasted 2–3 days, both years of the trial. The foraging activity of the hoverflies was maintained throughout the flowering period of the crop. The final emergence of pupae was checked at the end of the trial, obtaining values between 93 and 98% (without differences between treatments), thus reaching the desired target density. A treatment without pollinators was not contemplated in this trial because this crop requires pollen transport between the different flowers to obtain fruit set.

The pollination trial was repeated for two consecutive years, 2020 and 2021, in the same greenhouse. The pollinating syrphids were always introduced when the first pistillate flowers emerged, approximately 40 days after transplanting. The releases took place on 4 May 2020, and 19 April 2021, respectively. In each enclosure, water drinking troughs and sugar plates were placed to avoid premature mortality of the syrphids.

The temperature and relative humidity during both seasons of flowering were recorded using a datalogger (ORIA WA64). In 2020, the average temperature was 25.3 ± 8.4 °C, the maximum was 45.8 °C, and the minimum was 8.4 °C. In 2021, lower temperatures were recorded, with an average of 23.3 ± 8.1 °C, a maximum of 43.0 °C, and a minimum of 8.1 °C. Regarding relative humidity, in 2020 the average RH was 65.3 ± 20.6%, the maximum was 99.9%, and the minimum 21.4%. In 2021, the average was 72.5 ± 18.8%, the maximum 99.0%, and the minimum 28.9%.

### 2.2. Floral Visits

In 2020, the number and duration of floral visits in the triploid variety were specifically recorded because of their relevance compared to the diploid pollen-fertile variety. For these observations, two pistillate and two staminate triploid flowers were randomly selected each day in each cage. This procedure was repeated on three non-consecutive days. A total of 144 flowers (72 pistillate and 72 staminate flowers) were thus monitored for three minutes each. The observation took place between 10:00 and 12:00. The visit was considered legitimate only when the proboscis of the syrphid came into contact with the reproductive parts of the flowers for more than two seconds.

### 2.3. Pollen–Pistil Interaction

**Figure 1:**
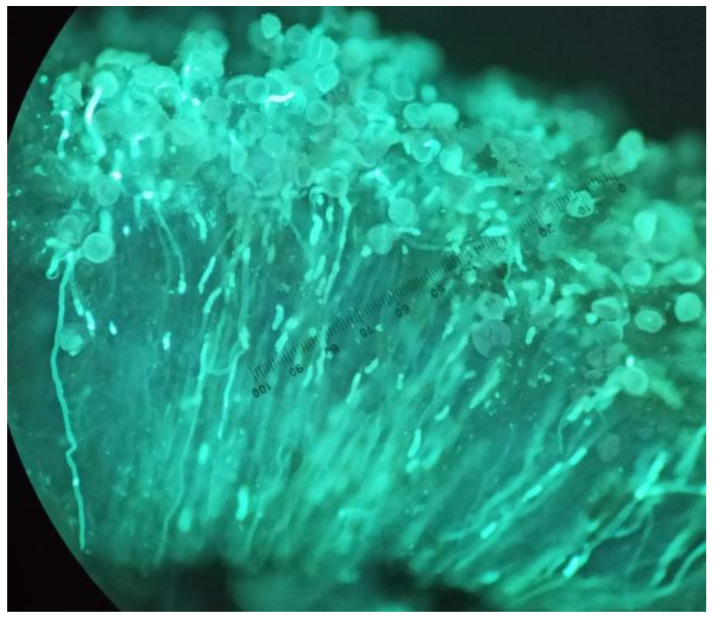
Adhesion of pollen grains and growth of pollen tube on watermelon stigma and style. Capture under epifluorescence microscopy (Nikon E600, ×20).

Pollen–pistil interaction was also analyzed in the triploid cultivar. For this purpose, the styles and stigmas were collected from 3 flowers in each cage and repeated on 3 non-consecutive days throughout the flowering period of both years, for a total of 216 flowers, 108 per year. Flowers were randomly tagged and collected 48 h after anthesis. The flowers were then preserved in FAA (formalin, glacial acetic acid, and 70% alcohol, in a 1:2:17 *v*/*v* ratio) before analysis under epifluorescence microscopy (Nikon E600, ×20) after staining them with aniline blue [42] to quantify pollen adhesion on the stigma (Figure 1).

**Figure 2:**
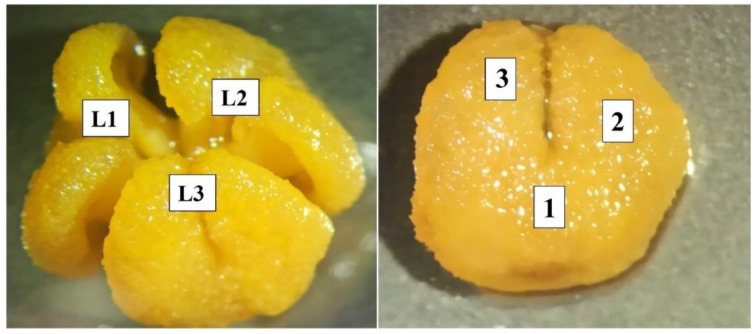
The three stigmatic lobes (L1, L2 and L3) of the watermelon flower (**left**) and the sampling areas in each lobe (1, 2 and 3 points) of the watermelon flower (**right**).

In this sense, the number and distribution of pollen grains adhered to the stigma in the three lobes of each flower were estimated by counting the number of pollen grains in three areas of each of the three stigma lobes of the watermelon flower (Figure 2). Subsequently, the total number of pollen grains per flower was calculated considering the mean stigma size, calculated as 25.80 mm^2^ for each lobe (Figure 2). The variability of pollen adhesion among stigma lobes was also estimated for each treatment to take into account the importance of an even distribution of pollen on the stigma for the production of symmetrical commercial fruit. The percentage of flowers with attached pollen was also determined.

### 2.4. Fruit Set, Yield and Components

The pollinating efficiency of the hoverfly *E. aeneus* was evaluated by comparing the effects of the three release densities on fruit set and yield in both the diploid and the triploid cultivar. In addition, earliness was determined by following the number of fruits setting in the three first weeks after blooms in each replicate. The number of fruits of each replicate was assessed by counting all ripe fruits at harvest. The weight of each fruit was determined in the field with a portable scale. Finally, the fruits were categorized according to the Commission Regulation (EC) No. 1862/2004 of 26 October 2004, which establishes the marketing standard for watermelon in the European Union. If the triploid watermelons were not deformed, and were uniform in color and weighed over 2 kg, they were classified as Category I fruit. Category II included slightly deformed fruits or fruits weighing less than 2 kg. Fruits not included in the first two categories were classified as non-commercial, and corresponded to cracked or excessively deformed fruit weighing less than 1 kg. In the case of diploid fruit, since they are of a “mini” variety, the minimum weight established for Category I was 1.5 kg, and for Category II was 1 kg, with the remaining fruit being discarded.

The number of seeds per fruit was estimated in five diploid and five triploid watermelon fruits per cage by counting the seeds in ¼ of the fruit. The seeds of diploid watermelons are easily recognizable, as they are fully developed and have a dark color. However, in the triploid cultivar, white, smaller, and tender seeds made counting more difficult.

Finally, soluble solids content (°Brix) was determined in five diploid and five triploid fruits per treatment and repetition, in both years. For this purpose, an ATAGO PR-101α digital refractometer was used, taking the average value of three recordings taken from the pulp of each processed fruit.

### 2.5. Statistical Analysis

Statistical analysis was performed with R Statistical software (R-Core Team, version 4.0.2, Vienna, Austria). Generalized Linear Models (GLMs) were used for insect activity analyses represented by the number and duration of flower visits with a previous square root transformation on the dependent variables to ensure a normal distribution of the response. Final models were selected by comparing the Akaike Information Criterion (AICc) value versus the value of the initial full models. Subsequently, means were separated using Tukey’s test (*p* < 0.05). Welch’s t-test was used to compare the number of visits recorded in both types of flowers to determine the existence of significant preferences in the number of visits received in each of the three release densities.

For pollen–pistil interactions, the analysis of pollen adhered for each year was performed using a multifactorial ANOVA, with the dependent variable being the number of pollen grains attached to the stigma and the independent factor the release density. A GLM was developed to determine the appearance of differences in the number of pollen grains attached to the different lobes of each flower using the number of pollen grains as the dependent variable and the treatment and lobes as independent variables. After angular transformation, the percentage of flowers with more than 500 pollen grains attached was compared by ANOVA. Subsequently, means were separated using Tukey’s test (*p* < 0.05).

Fruit number and weight, yield, number of seeds, and soluble solids content were analyzed each year by ANOVA to compare the different release densities. In addition, commercial categorization was compared through the percentage of fruits after angular transformation. When significant differences were detected, means separation was performed by Tukey’s test (*p* < 0.05). Finally, a theoretical economic yield (EUR/ha) was calculated considering the sale price by farmers in Andalusia (Spain) in June 2020 and 2021, according to the production of each category (Table S1) [43]. The economic analysis does not include the cost of syrphids release because their industrial production is still under development and the selling price for large-scale crops is not currently available.

## 3. Results

### 3.1. Floral Visits

**Figure 3:**
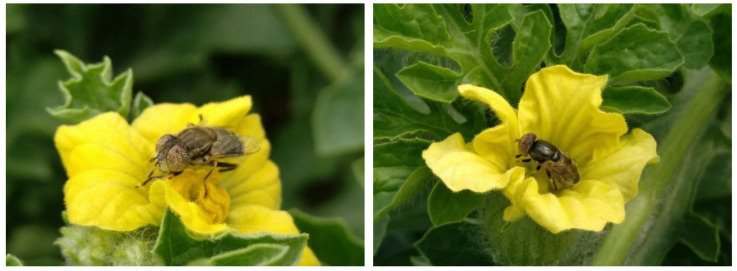
Floral visit of a female of *E. aeneus* on a pistillate watermelon flower (**left**), and floral visit of a male of *E. aeneus* on a staminate watermelon flower (**right**).

During 2020, 144 triploid watermelon flowers were observed, in which a total of 451 visits were recorded: 213 in pistillate flowers and 238 in staminate ones. Therefore, this pollinator visited both types of flowers in a similar number (Figure 3).

The number of floral visits made by *E. aeneus* was significantly higher as release density increased in both types of flowers (*p* < 0.0001) (Table 1). In pistillate flowers, a minimum of one visit was observed per 3 min of recording under LD, while a maximum of five were observed for both HD and MD. In staminate flowers, a minimum of one visit per 3 min was recorded in LD and a maximum of eight in HD (Table 2). There were also significant differences in the number of visits to pistillate versus staminate flowers in the HD treatment, where the number of visits to staminate flowers was significantly higher than the visits to pistillate flowers (t = −2.28; df = 41.41; *p* = 0.0281). However, these significant differences did not appear in MD (t = −0.17; df = 45.88; *p* = 0.8654) or LD (t = −1.42; df = 44.56; *p* = 0.1641) (Table 2). Regarding the duration of the floral visits, no differences were found either between flower types or due to release densities (Table 1). The minimum duration of the visits was five seconds, while the maximum duration recorded exceeded the observation time of 180 s.

### 3.2. Pollen–Pistil Interaction

Both the release densities and the year of the experiment significantly influenced the total number of pollen grains attached to the stigma of watermelon flowers (*p* < 0.0001, and *p* = 0.0045, respectively), but not their distribution among the stigma lobes of the flower. The highest number of pollen grains attached to the stigma was observed in HD in both years of the trial, followed by MD and LD (Table 3). Both HD and MD treatments showed optimal values of pollen adhesion, exceeding on average the threshold of 500 grains per flower (Table 3).

Release density had a positive effect on the percentage of flowers with more than 500 pollen grains adhered to the stigma in both 2020 (F = 10.81; *p* = 0.0040) and 2021 (F = 11.47; *p* = 0.0033). The highest percentage of flowers reaching this minimum value of adhered pollen was obtained in both years for HD treatment (91.8% and 69.8%), followed by MD (75.3% and 49.8%) and then by LD (38.8% and 27.5%). In this regard, in 2020, the percentage of total flowers with between 500 and 1000 pollen grains attached to the stigma was 45.4%, while 23.2% had more than 1000 pollen grains. In 2021, the percentage of flowers with between 500 and 1000 pollen grains attached to the stigma dropped to 34.3%, and only 14.8% had more than 1000 pollen grains. Some pollen adhesion took place on all sampled flowers, except for one single flower under LD treatment in 2021.

The zone of the stigma lobe (F = 7.78, *p* = 0.0010) and the release density (F = 4.64, *p* = 0.0131) influenced the amount of pollen adhered. Similarly, a gradient in pollen deposition within the stigma lobes was observed. The amount of pollen was much higher in the outermost zones than near the ovary (F = 162.52; *p* < 0.0001). The distribution of pollen grains among the different zones of the stigma lobes is shown in Table S2. Regarding the pattern of pollen distribution among stigma lobes of the same flower, no differences were found in either 2020 (F = 0.044; *p* = 0.9570) or 2021 (F = 0.420; *p* = 0.7951).

### 3.3. Fruit Set, Yield and Components

**Figure 4:**
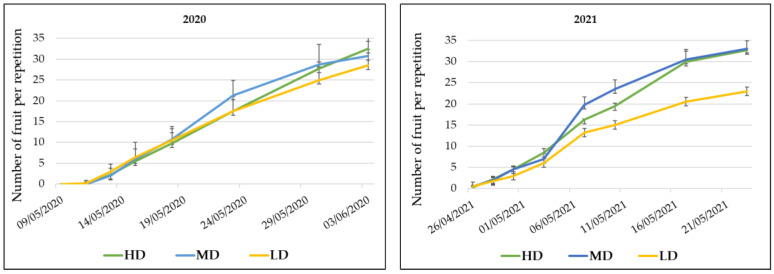
Cumulative production (diploid and triploid combined) as the number of fruit set throughout the watermelon cycle within the enclosures. Years 2020 (**left**) and 2021 (**right**) for the tree treatments (HD, MD, and LD release density).

The first fruit setting was observed one week after syrphids release; that is, approximately four days after adult emergence. Although a good fruit set was observed for LD, the highest number of fruits was recorded for HD and MD treatments, with remarkable differences with respect to LD in 2021 (Figure 4).

In 2020, the number of triploid fruits was significantly higher in HD than in LD treatment (F = 6.17; *p* = 0.0206), with the MD treatment in an intermediate position. There was no significant difference in the production of diploid fruits among treatments (F = 0.64; *p* = 0.5483). In 2021 there was again a greater number of triploid fruits in HD, followed by MD and LD (F = 7.40; *p* = 0.0126), and no effect for diploid fruits (F = 1.91; *p* = 0.2041) (Table 4). Higher release density also had positive effects on fruit weight, indicating a progressive response to increased pollen deposition. In 2021, significant differences in response to release density were observed in the weight of the triploid fruits (F = 5.35; *p* = 0.0295), but not in diploid fruits (F = 0.08; *p* = 0.9256) (Table 4). Similarly, crop yield showed significant differences according to release density for the triploid cultivar in both 2020 (F = 4.47; *p* = 0.0449) and 2021 (F = 24.92; *p* = 0.0002), but not for the diploid cultivar either in 2020 (F = 0.43; *p* = 0.6609) or in 2021 (F = 0.79; *p* = 0.4843) (Table 4).

**Figure 5:**
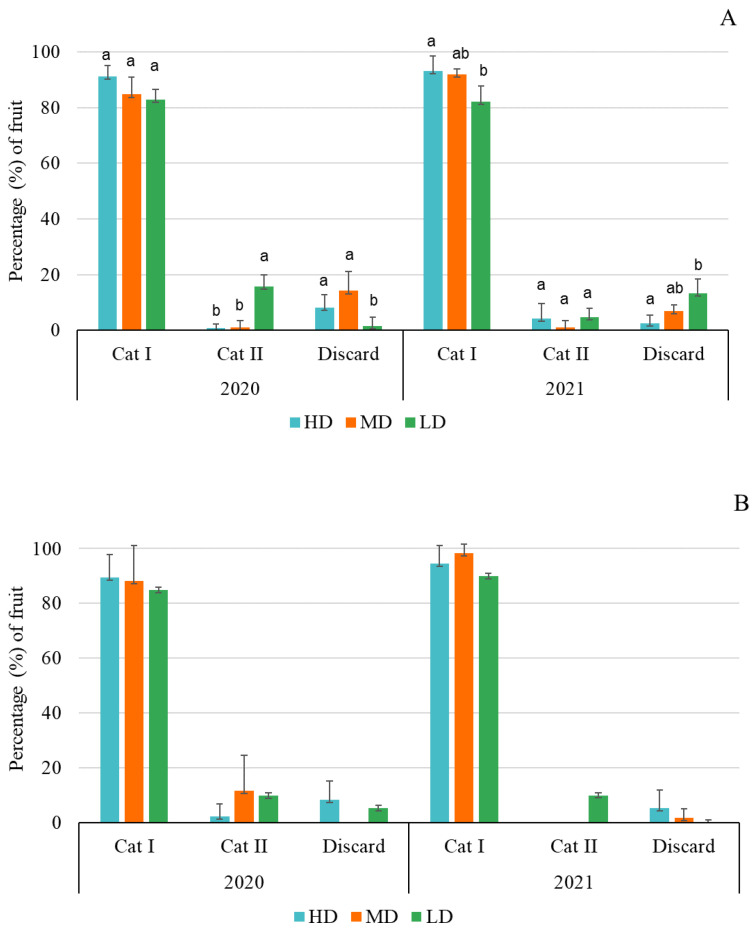
Percentage of triploid (**A**) and diploid (**B**) fruits according to quality categories and release density treatments (HD, MD, and LD). Different letters in each category for both cultivars indicate significant differences. (Tukey’s test, *p* < 0.05).

Regarding the categorization, the triploid fruits were primarily first class in both years. In 2020, there were no significant differences in the percentage of Category I fruits among the different release densities (F = 3.93; *p* = 0.0593), but a higher percentage of fruits in Category I were produced in HD (91.1%), followed by MD (84.7%), with LD as the least interesting treatment (82.7%). These differences did reach statistical significance for Category II (F = 22.68; *p* = 0.0003), with an inversely higher percentage of Category II fruit in LD (15.7%) compared to MD (1.1%) and HD (0.8%). The percentage of non-commercial fruits was also significantly related to release density in 2020 (F = 5.89; *p* = 0.0231), being higher in MD (14.2%) and HD (8.1%) versus LD (1.6%) (Figure 5A). In 2021, for Category I fruits, variations among treatments were significant (F = 4.67; *p* = 0.0406). A significantly higher percentage of fruits was achieved with HD (93.6%) versus LD (82.0%) (Figure 5A). Differences were not significant in Category II fruits (F = 0.96; *p* = 0.4189). Finally, the percentage of discarded fruits also depended on the density of release (F = 7.57; *p* = 0.0118), which was higher in LD this second year (Figure 5A).

In the diploid variety, in 2020, no significant differences were observed among the different release densities for Category I fruits (F = 0.10; *p* = 0.9076), Category II (F = 0.97; *p* = 0.4142), and discard (F = 2.90; *p* = 0.1018) fruit. The same occurred in 2021, when no significant differences were found among treatments in the percentage of Category I fruits (F = 0.25; *p* = 0.7844), Category II fruits (F = 1.00; *p* = 0.4053), and discard (F = 1.53; *p* = 0.2672) fruits. In all syrphid densities, the highest percentage of diploid fruits corresponded to Category I in both years (Figure 5B).

The number of seeds in diploid fruits was not significantly influenced by pollinator release density, either in 2020 (F = 1.71; *p* = 0.2343) or in 2021 (F = 3.16; *p* = 0.0915). However, seed number increased with release density in the triploid cultivar in both 2020 (F = 11.46; *p* = 0.0034) and 2021 (F = 13.18; *p* = 0.0021). Soluble solids content was independent of pollinator release density, with values of °Brix ranging from 6.3 to 11.7 in 2020 and from 9.7 to 14.4 °Brix in 2021 for diploid fruit. In the case of triploid fruits, °Brix values were between 9.2 and 14.0 in 2020 and between 10.2 and 16.0 °Brix in 2021 (Table 5).

Finally, the commercial yield was greatly enhanced by increasing the release density of the syrphid, as was the economic return for the grower. Higher yields and profits were obtained for the triploid variety under HD treatment, followed by MD and LD in both years (Table 6).

## 4. Discussion

Deficient pollination services negatively affect watermelon yield due to a deficit in pollen transfer to pistillate flowers [10]. In this trial, *E. aeneus* showed an important effectiveness, with an optimum yield achieved in both years with a high number of heavy fruits per plant, reaching yields between 30 and 55 t/ha (Table 6), and most of the fruit belonging to Category I (Figure 5). According to several authors, watermelon yield under greenhouse conditions in Spain are between 4 and 6 kg/m^2^ when pollinated by honey bees and/or bumble bees (no details on cultivars, ratios, planting density or insect pollinators given) [43,44,45], values similar to those obtained here. Therefore, the implementation of these eristaline syrphids as managed pollinators offers the advantage of providing an easy and safe method for farmers since they are not stinging or aggressive insects. 

Since triploid plants practically do not produce viable pollen, orchard design requires 20–33% diploid pollen donor plants [46,47], ranging from 1:2 to 1:5 in outdoor crops [3]. Greenhouse watermelon production systems are generally designed with ratios of 1:2 or 1:3, as in this trial. Higher yields can be expected for the triploid cultivar with a 1:2 ratio of diploid variety, due to the increased availability of viable pollen. Other techniques consisting of disposable pollinizers should also be evaluated for higher yields.

The number of pollinators required in protected watermelon crops has been established between two or three honey bee hives or seven bumble bee hives per hectare [8,43]; this means densities close to 20 individuals/m^2^, depending on the variation in the number of honey bee workers in the hive [48]. Our results show the effectiveness of *E. aeneus* as pollinator and also the positive effect of increasing release density, with higher yields (23.8 to 41.8% in 2020 and 36.3 to 46.7% in 2021) for the triploid cultivar at densities of 30 and 45 individuals/m^2^, respectively (Table 4). On the contrary, density release above 15 individuals/m^2^ did not increase the yield of the diploid watermelon cultivar. The proximity of pollen donors and recipient flowers in the diploid cultivar could reduce the energetic cost of the pollinator flight [49], achieving good pollination levels even with fewer pollinating insects. Release densities evaluated in this trial require confirmation on a larger scale for both cultivars. A larger flight space could favor greater pollen dispersal and thus reduce the number of individuals required. 

Factors affecting pollination in watermelon include the number of visits the flowers receive, the number of floral visits performed during the short period of floral opening, and the even distribution of pollen grains on the stigma. In this trial, the foraging activity of *E. aeneus* throughout the day was not analyzed but the observations made in parallel watermelon trials, and other crops, confirm that the main period for feeding on pollen and nectar is in the morning [50]. This higher number of floral visits during the morning is related to higher fertilization of watermelon flowers, due to a higher stigmatic receptivity [3]. It is worth noting that *E. aeneus* shows no preference for watermelon flower type, unlike the honey bee, which more assiduously visits the staminate flowers [33,36]. *E. aeneus* visitation patterns are extremely important in monoecious crops, such as watermelon, since the plants require visits to both flower sexes, and especially first to staminate flowers bearing viable pollen [51,52].

Pistillate flowers of watermelon require multiple insect visits to ensure high levels of fertilization and good seed and fruit set. Previous work with hymenopterans suggests a highly variable range of visits, between 10 and 60, to ensure a successful fruit set and commercial fruit quality [8,53,54,55]. In this trial, rates of between 33 and 78 visits/h to pistillate flowers were recorded depending on the density of hoverfly release, and between 38 and 93 visits/h for staminate flowers (Table 2). The higher density of hoverflies did not modify the duration of the visits, either to staminate or to pistillate flowers; however, it was observed that this significantly increased the number of visits received by both types of flowers (Table 2). The duration of visits is related to pollination efficiency since longer visitation time and consequent movements within the pistillate flower facilitate greater and better distribution of pollen grains on the stigma [56]. Our results show an average duration of 45 s per flower, a longer duration than the duration recorded for bees, and with no differences according to flower type (Table 2). In a parallel experiment, we recorded floral visits by bees of 6 s on staminate flowers and 12 s on pistillate flowers, like the values recorded by Araújo [57]. Garantonakis [27] found floral visits of 57 s for mining bees and 19 s for honey bees in watermelon, and Njoroge [33] found even shorter durations.

If the duration of the visits resulted in the same, then the deposition of pollen grains on the flowers will depend more on the number of visits and the transport capacity of the insects. In watermelon, a minimum of 500–1000 pollen grains on stigma is necessary to obtain commercial fruits in diploid cultivars [5,8,9]. For this 2020 experiment, 91.7% of flowers reached this minimum of 500 pollen grains in HD, 75.0% in MD, and 38.9% in LD. In 2021, this percentage of flowers was lower, with 69.4% for HD, 50.0% for MD, and 27.8% for LD. Crop management was similar in both years, and no additional problems were detected that could explain a decrease in the number of pollen grains adhered. As observed by other authors, the difference found in each of the years could have been due to spatiotemporal variations in pollinator efficacy [28]. However, in both years, the higher release density allowed a majority of flowers with the minimum number of pollen grains required to be reached. This greater adhesion of pollen grains led to an increased number of seeds per fruit (Table 5). Similarly, a homogeneous distribution of pollen grains was observed in the three stigmatic lobes of the flower, independently of the total number of deposited pollen grains or the release density of hoverflies. This is essential for the optimal development of this multi-seeded fruit with symmetric shape and adequate size [3,58]. It was also observed that the highest percentage of pollen grains was found in the outermost parts of the flower stigma, coinciding with areas of easy access for pollinators, compared to areas near the ovary, which are more difficult zones for the pollinator to access.

According to prediction models, when compared to smaller insects, honey bees and bumble bees deposit more pollen grains per day (4335 and 2814, respectively) [55]. In this regard, our results show that the total quantity of pollen deposited on flowers by *E. aeneus* was lower than the estimates of this model for honey bees and bumble bees, but higher than for wild bees (Table 3) [30,55]. This suggests that the larger size of honey bees and bumble bees favors greater pollen deposition. Therefore, a higher number of *E. aeneus* visits could be required given its smaller size. Nonetheless, recent studies carried out on watermelon showed no significant differences in the total number of pollen grains deposited and adhered to the stigma between honey bees and *E. aeneus* (unpublished data).

Finally, the ‘most effective pollinator principle’ states that floral traits are shaped by pollinators that visit the flower most frequently and efficiently [59,60]. Efficacy is defined as the probability that pollen is transferred from the anthers to the pollinator, the probability that the pollinator will visit another flower of the same species before the pollen is lost, and the probability of pollen being transferred from the pollinator to the stigma of the new flower. In this case, *E. aeneus* collected viable pollen from the anthers of staminate flowers, frequently visited pistillate flowers, and regularly deposited and distributed viable pollen on the stigma of these flowers.

## 5. Conclusions

This work concluded that *E. aeneus* is an effective pollinator for the protected cultivation of diploid and triploid watermelon. The most effective release density for these conditions is between 30 and 45 individuals/m^2^. In the case of diploid production, the release density can be reduced to between 15 and 30 individuals/m^2^.

## Figures and Tables

**Table 1:** Generalized linear models (deviance analysis, type III) for the number and duration of the visits recorded on watermelon triploid flowers. The independent variables were release density (15, 30 and 45 individuals/m^2^) and flower type (staminate versus pistillate).

Parameters	Variable	SS	Df	F-Value	Pr (>F)
Number of visits	Release density	13.99	2	108.361	<0.0001 ***
	Type of flower	0.32	1	4.971	0.0274 *
	Residuals	9.04	140		
Duration of visits	Release density	3.41	2	0.346	0.7079
	Type of flower	3.84	1	0.779	0.3779
	Residuals	2222.22	451		

SS: Sum of Squares; Df: degrees of freedom; Pr (>F): *p*-value associated with the value in the F-value column. Statistical significance of variables (release density and type of flower) for each parameter is represented without * for non-significant (*p* > 0.05), * for *p* < 0.05, *** *p* < 0.001.

**Table 2:** Number (n) and duration (seconds) of flower visits (mean ± SE) made by the syrphids on both types of flowers, at each treatment (release density: HD, MD and LD), during 3 min of observation.

Treatments	Number of Visits (n)		Duration of Visits (s)	
	Pistillate Flower	Staminate Flower	Pistillate Flower	Staminate Flower
HD	3.92 ± 0.18 a	4.63 ± 0.25 a	40.51 ± 2.54	42.63 ± 3.24
MD	3.33 ± 0.18 b	3.38 ± 0.17 b	49.74 ± 4.29	42.08 ± 4.04
LD	1.63 ± 0.13 c	1.92 ± 0.16 c	45.28 ± 4.84	40.67 ± 4.76

Different lowercase letters in the same column indicate significant differences among treatments (release densities). Mean separation by Tukey’s test at *p* < 0.05.

**Table 3:** Number of pollen grains adhered (mean ± SE), minimum and maximum values between parentheses, on the stigma of triploid watermelon flowers for each treatment (release density: HD, MD and LD), in both years of the trial.

Treatment	Year 2020	Year 2021
HD	904.71 ± 109.10 a (290–1819)	716.95 ± 24.32 a (233–1804)
MD	811.88 ± 57.05 a (36–1763)	555.56 ± 34.25 a (9–1167)
LD	439.38 ± 64.16 b (36–1101)	348.30 ± 78.39 b (0–1278)

Different lowercase letters in the same column indicate significant differences between release densities. Mean separation by Tukey’s test at *p* < 0.05.

**Table 4:** Number of fruits per plant, fruit weight, and yield in the enclosures, for both watermelon cultivars. Years 2020 and 2021 for the three treatments (release density: HD, MD and LD).

Parameters	Treatment	Year 2020		Year 2021	
		Diploid	Triploid	Diploid	Triploid
Fruit per plant (num)	HD	6.17 ± 0.33 a	4.89 ± 0.77 a	7.25 ± 0.83 a	3.71 ± 0.22 a
	MD	5.38 ± 0.43 a	3.67 ± 0.31 ab	6.25 ± 0.59 a	3.54 ± 0.14 a
	LD	6.50 ± 1.14 a	2.79 ± 0.36 b	5.00 ± 0.98 a	2.71 ± 0.22 b
Fruit weight (kg)	HD	3.06 ± 0.38 a	5.24 ± 0.20 a	2.81 ± 0.21 a	5.50 ± 0.23 a
	MD	3.02 ± 0.09 a	5.02 ± 0.35 a	2.91 ± 0.10 a	4.89 ± 0.22 ab
	LD	2.99 ± 0.21 a	5.00 ± 0.22 a	2.92 ± 0.29 a	4.18 ± 0.37 b
Yield (kg/m^2^)	HD	4.63 ± 0.49 a	5.93 ± 0.72 a	4.99 ± 0.35 a	5.07 ± 0.26 a
	MD	4.07 ± 0.37 a	4.67 ± 0.64 ab	4.51 ± 0.33 a	4.31 ± 0.05 a
	LD	4.77 ± 0.76 a	3.45 ± 0.33 b	3.84 ± 1.03 a	2.82 ± 0.30 b

Different lowercase letters in the same column and parameter indicate significant differences between release densities. Mean separation by Tukey’s test at *p* < 0.05.

**Table 5:** Number of seeds in 1/4 of the fruit and soluble solids content (°Brix) in diploid and triploid watermelon cultivars for both years for the three treatments (release density: HD, MD, and LD).

Parameters	Treatment	Year 2020		Year 2021	
		Diploid	Triploid	Diploid	Triploid
Number of seeds per ¼ fruit	HD	346.75 ± 18.25 a	311.75 ± 13.94 a	424.50 ± 29.24 a	434.50 ± 19.21 a
	MD	342.00 ± 12.89 a	304.75 ± 9.68 a	403.00 ± 19.00 a	400.50 ± 23.29 a
	LD	314.75 ± 4.82 a	243.00 ± 9.31 b	350.00 ± 13.46 a	289.00 ± 20.17 b
°Brix	HD	9.17 ± 0.32 a	11.09 ± 0.17 a	11.62 ± 0.34 a	13.08 ± 0.40 a
	MD	9.45 ± 0.36 a	11.49 ± 0.28 a	11.90 ± 0.19 a	12.75 ± 0.21 a
	LD	9.42 ± 0.74 a	11.25 ± 0.29 a	11.89 ± 0.39 a	12.43 ± 0.26 a

Different lowercase letters in the same column and parameter indicate significant differences between release densities. Mean separation by Tukey’s test at *p* < 0.05.

**Table 6:** Commercial yield (t/ha) and economic return (EUR/ha) of watermelon orchard adding production of both cultivars. Years 2020 and 2021 for the three treatments (release density: HD, MD, and LD).

Parameters	Treatment	Year 2020			Year 2021		
		Diploid	Triploid	Total 2020	Diploid	Triploid	Total 2021
Yield (t/ha)	HD	15.25	39.44	54.69	16.12	33.57	49.69
	MD	13.55	30.13	43.68	14.83	28.11	42.94
	LD	15.65	22.96	38.61	12.79	17.91	30.70
Economic yield (EUR/ha)	HD	2877.89	12,118.11	14,996.00	2740.44	8232.24	10,972.69
	MD	2489.82	9259.15	11,748.97	2520.88	7001.28	9522.16
	LD	2880.50	6604.32	9484.82	2163.24	4356.88	6520.13

## Data Availability

The data that supports the findings of this study are available on request from the author.

## Supplementary Materials

The following supporting information can be downloaded at: https://www.mdpi.com/article/10.3390/insects13111021/s1, Table S1: Sales price (EUR/kg) by farmers of diploid and triploid watermelon cultivar in Andalusia (Spain), according to fruit category, for June 2020 and 2021; Table S2: Ranges of percentages of pollen grains attached to the stigmatic lobe of watermelon flowers according to observation zone (outermost, intermediate and innermost) for both years of the trial and treatments (release density: HD, MD and LD).
