# Supplementary material for: Pollination Effectiveness of the Hoverfly Eristalinus aeneus (Scopoli, 1763) in Diploid and Triploid Associated Watermelon Crop
Source: Insects. 2022 Nov 5;13(11):1021. doi: 10.3390/insects13111021 (PMC9697508; doi:10.3390/insects13111021)
Supplement: Supplementary file 1 [file insects-13-01021-s001.zip › insects-1969465-supplementary.pdf]

Table S1. Sales price (EUR/kg) by farmers of diploid and triploid watermelon cultivar in Andalusia (Spain), according to fruit category, for June 2020 and 2021.

Title 1	Category	Year 2020	Year 2021
Diploid	Category I	0.19	0.17
	Category II	0.08	0.14
Triploid	Category I	0.31	0.25
	Category II	0.07	0.07

Table S2. Ranges of percentages of pollen grains attached to the stigmatic lobe of watermelon flowers according to observation zone (outermost, intermediate and innermost) for both years of the trial and treatments (release density: HD, MD and LD).

Zone of stigmatic lobe	Year 2020			Year 2021		
	HD	MD	LD	HD	MD	LD
Outermost (3)	46 – 51%	46 – 54%	40 – 47%	35 – 53%	35 – 45%	36 – 49%
Intermediate (2)	32 – 37%	29 – 41%	35 – 38%	31 – 45%	32 – 40%	27 – 35%
Innermost (1)	17 – 18%	12 – 19%	15 – 24%	15 – 26%	35 – 45%	19 – 33%
